# Drug Screening in Human Cells by NMR Spectroscopy Allows the Early Assessment of Drug Potency

**DOI:** 10.1002/anie.201913436

**Published:** 2020-02-25

**Authors:** Enrico Luchinat, Letizia Barbieri, Matteo Cremonini, Alessio Nocentini, Claudiu T. Supuran, Lucia Banci

**Affiliations:** ^1^ CERM—Magnetic Resonance Center Università degli Studi di Firenze via Luigi Sacconi 6 50019 Sesto Fiorentino Italy; ^2^ Dipartimento di Scienze Biomediche Sperimentali e Cliniche “Mario Serio” Università degli Studi di Firenze Viale Morgagni 50 50134 Florence Italy; ^3^ Consorzio Interuniversitario Risonanze Magnetiche di, Metalloproteine Via Luigi Sacconi 6 Sesto Fiorentino Italy; ^4^ Dipartimento Neurofarba Sezione di Scienze Farmaceutiche Università degli Studi di Firenze Via Ugo Schiff 6 50019 Sesto Fiorentino Italy; ^5^ Dipartimento di Chimica Università degli Studi di Firenze Via della Lastruccia 3 50019 Sesto Fiorentino Italy

**Keywords:** drug design, drug screening, in-cell NMR spectroscopy, structural biology, sulfonamide

## Abstract

Structure‐based drug development is often hampered by the lack of in vivo activity of promising compounds screened in vitro, due to low membrane permeability or poor intracellular binding selectivity. Herein, we show that ligand screening can be performed in living human cells by “intracellular protein‐observed” NMR spectroscopy, without requiring enzymatic activity measurements or other cellular assays. Quantitative binding information is obtained by fast, inexpensive ^1^H NMR experiments, providing intracellular dose‐ and time‐dependent ligand binding curves, from which kinetic and thermodynamic parameters linked to cell permeability and binding affinity and selectivity are obtained. The approach was applied to carbonic anhydrase and, in principle, can be extended to any NMR‐observable intracellular target. The results obtained are directly related to the potency of candidate drugs, that is, the required dose. The application of this approach at an early stage of the drug design pipeline could greatly increase the low success rate of modern drug development.

## Graphical abstract

**In‐cell NMR spectroscopy** is used for drug screening in human cells. The approach is demonstrated by screening candidate drugs against intracellular carbonic anhydrase 2. The method allows the determination of drug permeability and intracellular binding specificity, which are related to drug potency, at an early stage of drug development and without recurring to enzymatic assays.

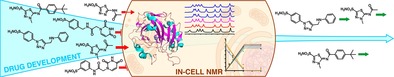

Rational drug design requires ligand‐based screenings and protein‐based structural studies to characterize the binding to the target protein, followed by lead optimization to increase the binding constant. The ability to reach intracellular targets must then evaluated with ad hoc cell‐based assays. Most drug candidates fail here or at later stages, due to lack of activity in cells or to the occurrence of adverse effects in vivo.^1^ Such failures are often linked to poor membrane permeability and/or to the lack of binding specificity in the cellular environment. Nuclear magnetic resonance (NMR) spectroscopy applied to living cells^2^, ^3^, ^4^, ^5^, ^6^ has the potential to overcome these critical bottlenecks, as it can directly observe macromolecule–ligand interactions at atomic resolution within the cellular environment.^2^, ^7^, ^8^ Herein, we report an approach to perform protein‐observed ligand screening by NMR directly in the cytosol of living human cells. The approach allows the assessment of membrane permeability and intracellular binding specificity of candidate drugs, which cannot be inferred from in vitro analysis, and can reveal interesting and strikingly different behaviour for compounds that have similar properties in vitro.

We applied the method to the screening of inhibitors of the second isoform of human carbonic anhydrase (CA2). In humans there are 15 CA isoforms,^9^ many of which are relevant drug targets involved in several pathologies.^10^, ^11^, ^12^, ^13^, ^14^ To date, several CA inhibitors are routinely administered in the treatment of glaucoma, epilepsy, or as diuretics,^15^, ^16^ with some of them in clinical development as antitumor agents,^17^, ^18^ In order to detect CA2 by NMR, the protein was directly expressed and labelled in the cytosol of human cells (see the Experimental Methods section of the Supporting Information).^19^, ^20^ NMR signals arising from [^15^N]‐CA2 were clearly detected in the ^1^H‐^15^N correlation spectra (Supporting Information, Figure S1), indicating that the intracellular protein is soluble and free from interactions with slow‐tumbling cellular components, which would otherwise increase transverse relaxation and cause signal broadening beyond detection.^21^ Signals from slow‐exchanging histidine side chain amide protons located in the active site were clearly detected in a background‐free region of the 1D ^1^H NMR spectrum between 11 and 16 ppm, allowing protein–ligand interactions to be monitored without the need for isotopic labelling (Supporting Information, Figure S2).

The interaction between intracellular CA2 and two approved drugs, acetazolamide (**AAZ**) and methazolamide (**MZA**), was monitored by ^1^H‐^15^N NMR. Spectra from cells expressing [^15^N]‐CA2 treated with excess of **AAZ** or **MZA** showed clear differences compared to untreated cells, indicating that both drugs had bound the intracellular protein (Figure 1 a,b). Inhibition of intracellular CA2 was confirmed by total CO_2_ hydration activity measured by stopped‐flow, which was largely decreased in lysates from cells expressing CA2 treated with inhibitor with respect to untreated cells (Supporting Information, Figure S3). In vitro, titration of CA2 with one equivalent of **AAZ** or **MZA** resulted in quantitative binding (Supporting Information, Figure S4), consistent with nanomolar dissociation constants (*K*_d_^AAZ^=12.5±1.0 nm; *K*_d_^MZA^=14±0.9 nm, see the Experimental Methods section in the Supporting Information). The protein–ligand interaction can be mapped at the single‐residue level by chemical shift perturbation (CSP) analysis. The high similarity between in‐cell and in vitro CSP plots revealed that both ligands bind to intracellular CA2 in an almost identical fashion as in vitro (Supporting Information, Figure S5), that is also consistent with the atomic structure of the protein–drug adducts reported previously (Figure 1 d).^22^

**Figure 1:**
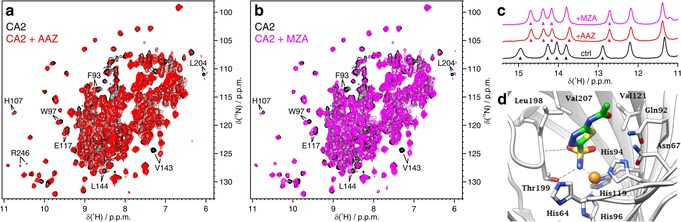
Overlay of ^1^H‐^15^N NMR spectra of cells expressing [^15^N]‐CA2 in the absence of ligands (black) and treated for 1 hour with a) 100 μm **AAZ** (red); b) 100 μm **MZA** (magenta). Some peaks shifting upon ligand binding are labelled. c) Imino region of the 1D ^1^H NMR spectra of unlabelled intracellular CA2 in the absence of ligands (black) and bound to **AAZ** (red) or **MZA** (magenta). Peaks used to obtain binding curved are marked with arrows. d) Active site view of CA2/**AAZ** complex (PDB 3HS4): the catalytic zinc ion (orange sphere) is located at the end of a large, conical cavity close to the protein centre and exhibits a tetrahedral coordination with three conserved histidine residues and, in the active form, a water molecule/hydroxide ion as fourth ligand.^9^ The latter is replaced by the deprotonated sulfonamide nitrogen in the enzyme/inhibitor adduct. The SO_2_NH^−^ moiety is additionally H‐bonded as donor to the OH moiety and as acceptor to the amidic NH of residue Thr199.

Binding of **AAZ** and **MZA** to unlabelled CA2 in the slow exchange regime was clearly observed in the imino region of the 1D ^1^H NMR spectra, both in cells and in vitro, due to the proximity of the observed nuclei to the ligand binding site (Figure 1 c,d and Supporting Information, Figure S6 a,b). Despite their known pH‐dependence, these nuclei proved to be excellent reporters of the free and bound states of CA2 (Supporting Information, Figure S6 c). The surprisingly good signal separation between free and bound states, despite the broader spectral lines of in‐cell NMR spectra, allowed the area under each peak to be retrieved by deconvolution analysis (Supporting Information, Figure S7). Simple 1D ^1^H NMR spectra could therefore provide a quantitative measure of the free and ligand‐bound fractions of intracellular CA2, providing a less time‐ and cost‐intensive strategy compared to 2D ^1^H‐^15^N NMR.

^1^H in‐cell NMR and deconvolution analysis were subsequently applied to screen a larger set of CA inhibitors, selected among recently reported sulfonamide‐derivatives, for which the binding affinity to CA2 had been previously characterized in vitro and ranged from low‐nanomolar to high‐micromolar (Figure 2).^23^, ^24^, ^25^ To establish whether this approach could discriminate ligands based on their cell permeability, a CA inhibitor (**C18**) that is unable to diffuse through the plasma membrane was also included. Strikingly, two categories of ligands emerged, that did not correlate with the differences in binding affinity: those that bind intracellular CA2 quantitatively, similar to **AAZ** and **MZA** (**1**, **3**, and **4**, Figure 3), and those for which binding is negligible, similar to **C18** (**2**, **5**, **6**, **7**, and **8**, Supporting Information, Figure S8). In comparison, ligand binding was clearly observed in vitro, both in the ^1^H‐^15^N and in the ^1^H NMR spectra (Supporting Information, Figure S9).

**Figure 2:**
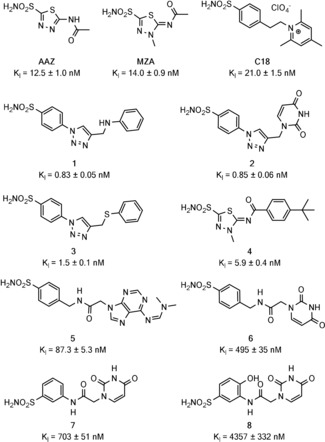
Sulfonamide‐derived CA inhibitors analyzed in this study. *K*_I_ measured in vitro for CA2 are reported (see the Experimental Methods section of the Supporting Information).

**Figure 3:**
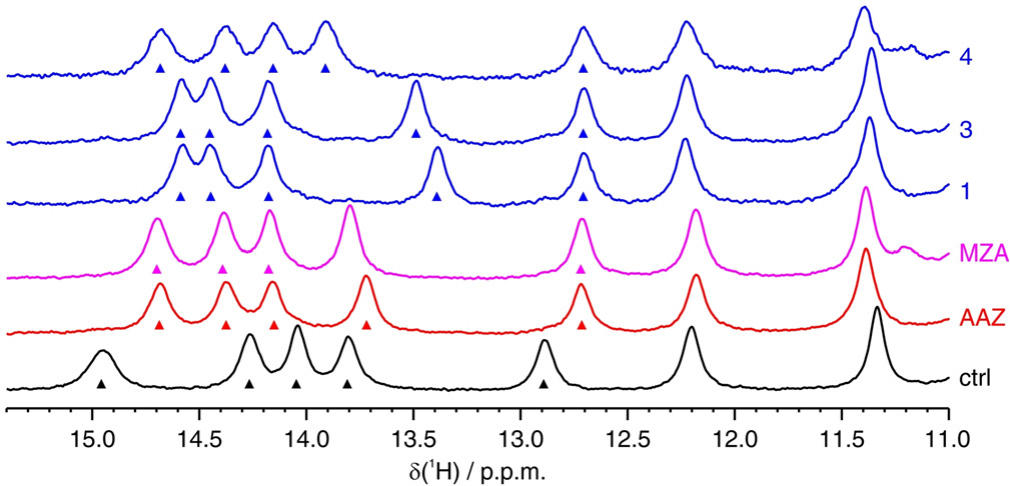
Imino region of the 1D ^1^H NMR spectra of cells expressing CA2 in the absence of ligands (black) and treated with 100 μm of each ligand for 1 hour, showing complete binding to intracellular CA2: **AAZ** (red), **MZA** (magenta) and ligands **1**, **3**, **4** (blue). Peaks used to obtain binding curves are marked with arrows.

Therefore, a simple high‐dose ligand screening by ^1^H in‐cell NMR could discriminate between “successful” and “unsuccessful” inhibitors without recurring to any cell‐based activity assay, by directly observing intracellular binding. However, no insights were provided on the reasons why some ligands, even those with high in vitro affinity, do not bind intracellular CA2 at all. While several phenomena could prevent a ligand from binding an intracellular target, the most likely reasons are a low permeability of the plasma membrane and a low binding specificity to the target, that is, the presence of other intracellular molecules with comparatively high affinity for the same ligand. To provide a mechanistic hypothesis on the behaviour of the “unsuccessful” ligands, the “successful” ligands were analysed by in‐cell NMR in a dose‐ and in a time‐dependent manner. First, cells expressing CA2 and treated for 1 hour with increasing concentrations of each ligand were analysed by ^1^H in‐cell NMR and spectral deconvolution. Except **AAZ**, all the ligands followed a common trend, showing incomplete binding at low doses and reaching complete binding at around 5–15 μm (Figure 4 a–e). At the lowest ligand concentrations, the incomplete binding may be due to a lower‐than‐one ligand to CA2 molar ratio. Surprisingly, **AAZ** resulted in a much shallower dose‐dependent binding curve, showing no binding below 10 μm and complete binding only around approximately 75 μm. Incomplete binding of **AAZ** at higher molar ratios suggests that it either diffuses slowly through the plasma membrane or strongly binds to other intracellular molecules. While competition binding is an equilibrium effect, slow diffusion kinetics should be observed in time‐dependent binding experiments at fixed ligand concentration. Indeed, treating the cells with either 27 μm **AAZ** or 2 μm **MZA** for increasing time periods resulted in clear time‐dependent binding curves (Figure 5). Dose‐ and time‐dependent curves could then be fitted with a kinetic model to obtain membrane permeability coefficients, revealing that **AAZ** diffuses approximately 12‐fold slower than **MZA** (Figure 4 f). As both molecules passively diffuse through the plasma membrane and do not rely on active transport,^26^ such strikingly different behaviour should eventually affect drug permeability in human tissues. Indeed, the observed difference is reflected in the pharmacokinetic properties of the two drugs, as the recommended dosage for **AAZ** in the treatment of glaucoma is approximately 10‐fold higher than that of **MZA**.^27^ Therefore, the kinetics of membrane diffusion can greatly affect the behaviour of different ligands, irrespective of their binding affinity for the intracellular target. Interestingly, all the “unsuccessful” ligands screened here share a nitrogenous base, either a uracil (**2**, **6**, **7**, and **8**) or adenine (**5**) as a common feature, and have a much lower predicted skin permeability than the “successful” ligands (Figure 4 f), thus suggesting that the lack of binding of the former is likely the consequence of an exceedingly slow diffusion through the plasma membrane.

**Figure 4:**
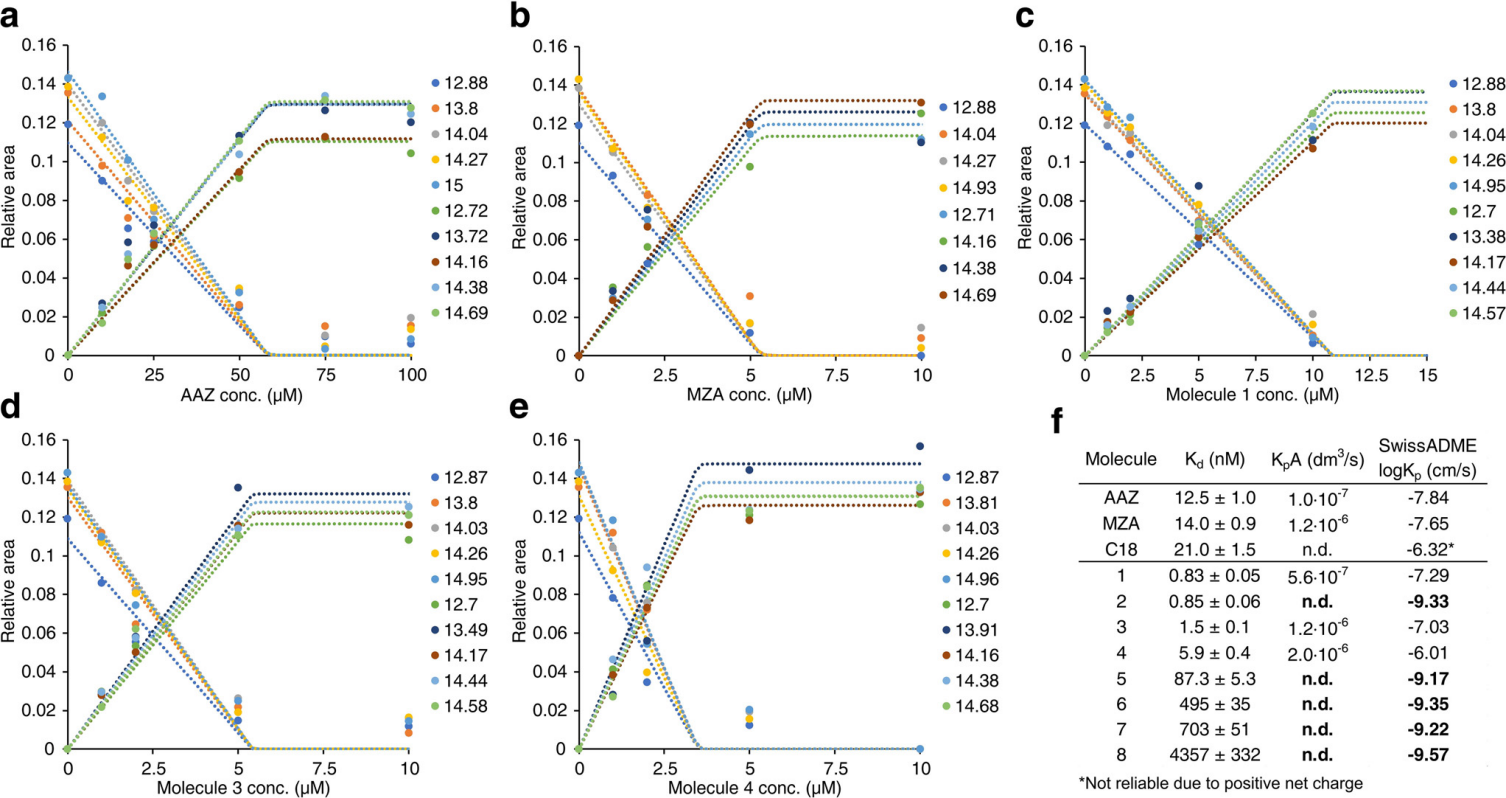
a–e) Dose‐dependent binding curves observed in cells expressing CA2 treated for 1 hour with different ligands at increasing concentrations, fitted with a time‐dependent binding equilibrium (See the Experimental Methods section of the Supporting Information). Each dot represents the area under a single peak in the imino region of the 1D ^1^H in‐cell NMR spectra, normalized to the total spectral area. f) Dissociation constant measured in vitro (*K*_d_), permeability coefficient × membrane area (*K*_p_ *A*) obtained from curve fitting and predicted skin permeability coefficient (log *K*_p_) for each molecule. “Unsuccessful” molecules correlate with lower skin permeability (shown in bold).

**Figure 5:**
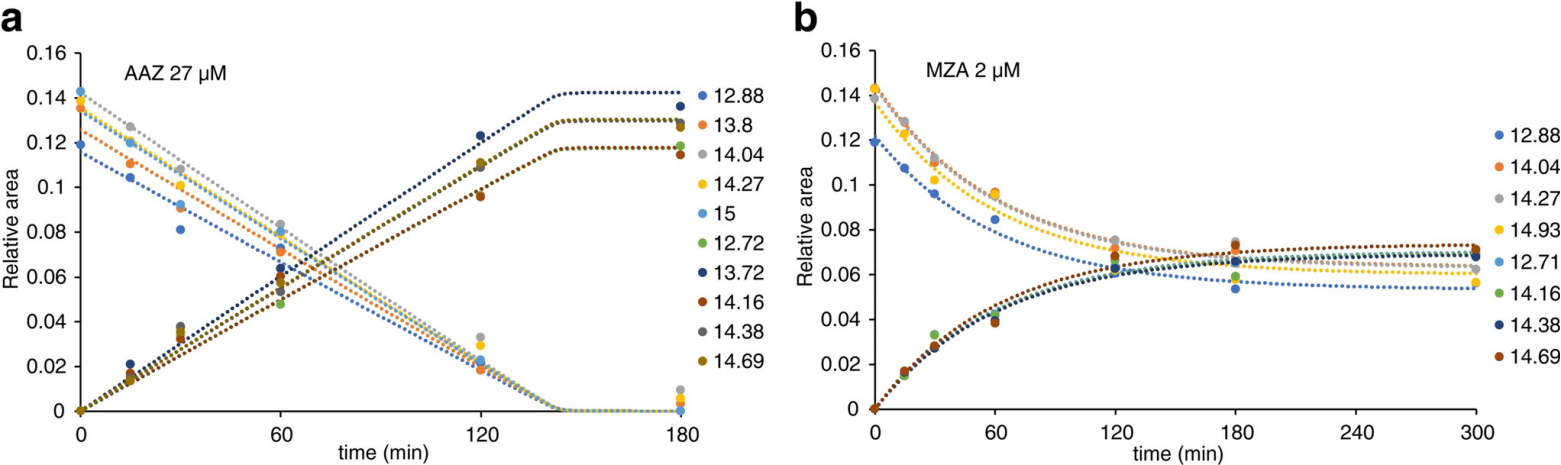
Time‐dependent binding curves observed in cells expressing CA2 treated with either 27 μm **AAZ** (a) or 2 μm **MZA** (b) for increasing time periods. Binding of **AAZ** was fitted with a time‐dependent binding equilibrium; binding of **MZA** was fitted with a time‐dependent diffusion in deficiency of external ligand with respect to the protein (See the Experimental Methods section of the Supporting Information). Each dot represents the area under a single peak in the imino region of the 1D ^1^H in‐cell NMR spectra, normalized to the total spectral area.

Once the cells are analysed under steady‐state conditions, that is, after the ligand had enough time to reach the intracellular target, the in‐cell binding curve can be fitted to obtain an apparent *K*_d_. Comparison with the *K*_d_ determined in vitro would reveal the extent of competition binding, thereby providing a measure of intracellular binding specificity. To obtain a meaningful apparent *K*_d_, the ligand to protein molar ratio should be higher than one. For this purpose, useful binding curves can be obtained from cells expressing the target protein at lower levels. Indeed, cells expressing an approximately 3‐fold lower level of CA2 and treated for 2 hours with concentrations of **MZA** ranging from 0.1 to 2 μm resulted in a steeper binding curve compared to cells with high levels of CA2, giving an apparent *K*_d_ similar, within the error, to that measured in vitro (Supporting Information, Figure S10 a,b). Interestingly, treatment with **4** under the same conditions resulted in an apparent *K*_d_≈20‐fold higher than in vitro, thus suggesting that other molecules may compete for binding **4** within the cell (Supporting Information, Figure S10 c,d). Therefore, quantitative binding curves obtained by NMR can provide meaningful information to describe the kinetics and thermodynamics of intracellular ligand binding.

Finally, to assess to which extent the “intracellular protein‐observed” approach can be generalized to other intracellular targets, ligand binding was tested on intracellular CA1. While CA1 reached lower expression levels than CA2, histidine amide protons were still detected in the 1D ^1^H NMR spectra, and free and bound species could be clearly separated (Supporting Information, Figure S11). Therefore, the approach should be applicable to other intracellular CA isoforms, which contain conserved zinc‐binding histidines, and in principle to any other protein that gives rise to ^1^H signals downfield of approximately 11 ppm^19^ or in any background‐free spectral region. More in general, by recurring to selective isotopic labelling strategies, such as amino acid type‐selective [^13^C]‐methyl or [^15^N]‐labelling, this approach can be applied to any soluble intracellular protein, provided that at least one signal is observable by in‐cell NMR and is sensitive to ligand binding.

Herein, we show that ligand screening can be performed in human cells towards a specific protein by “intracellular protein‐observed” NMR. Since the first proof‐of‐principle in human cells,^2^ in‐cell NMR application to protein‐observed ligand screening has been limited to bacteria.^28^ The approach shown here allows efficient in‐depth drug screening in human cells for assessing intracellular target‐binding capabilities, and also provides a way to characterize them in more quantitative terms, without recurring to chemical tagging of the protein.^29^ The currently low throughput of the approach can be greatly improved by increasing automation (for example, through the use of a temperature‐controlled NMR sample changer) and by screening multiple ligands simultaneously (for example, through matrix methods^28^). Importantly, this method does not rely on enzymatic activity measurements. Therefore, it provides a unique novel way to evaluate the effectiveness of drugs also against non‐enzymatic targets, each of them otherwise requiring indirect cell‐based assays to be established, such as cell proliferation, invasion, and viability/apoptosis assays. Ultimately, once framed within the modern drug development pipeline, this method could allow the assessment of the potency of a candidate drug, that is, the amount of drug required to exert an effect of given intensity, in a clinically relevant concentration range (0.1–100 μm). Such predictive ability would allow the optimization of potency at an earlier stage of the pipeline compared to cell‐ and animal‐based assays.

## Conflict of interest

The authors declare no conflict of interest.

## Supporting information

As a service to our authors and readers, this journal provides supporting information supplied by the authors. Such materials are peer reviewed and may be re‐organized for online delivery, but are not copy‐edited or typeset. Technical support issues arising from supporting information (other than missing files) should be addressed to the authors.

Supplementary
