# Supplementary material for: Drug Screening in Human Cells by NMR Spectroscopy Allows the Early Assessment of Drug Potency
Source: Angew Chem Int Ed Engl. 2020 Feb 25;59(16):6535–9. doi: 10.1002/anie.201913436 (PMC7187179; doi:10.1002/anie.201913436)
Supplement: Supplementary file 1 — Supplementary [file ANIE-59-6535-s001.pdf]

Angewandte Chemie
Eine Zeitschrift der Gesellschaft Deutscher Chemiker

Supporting Information

Drug Screening in Human Cells by NMR Spectroscopy Allows the Early Assessment of Drug Potency

*Enrico Luchinat, Letizia Barbieri, Matteo Cremonini, Alessio Nocentini, Claudiu T. Supuran, and Lucia Banci**

anie_201913436_sm_miscellaneous_information.pdf

Contents

1. Experimental Methods

  1.1. Gene cloning

  1.2. Human cell culture and transfection

  1.3. Protein quantification and intracellular distribution

  1.4. Treatment with inhibitors

  1.5. Stopped-flow measurements

  1.6. Expression and purification of recombinant CA2

  1.7. In vitro NMR experiments

  1.8. In-cell NMR experiments

  1.9. NMR data processing and signal deconvolution

  1.10. Binding curve fitting

2. Supplementary Figures

1. Experimental Methods

1.1. Gene cloning

To generate the mammalian expression plasmids, the cDNAs encoding full-length human CA2 (amino acids 1–260, GenBank: NP_000058.1) and human CA1 (amino acids 1–261, GenBank: NP_001122303.1) were amplified by PCR and cloned into the pHLsec vector[1] between EcoRI and XhoI restriction enzyme sites (CA2) and between HindIII and XhoI restriction enzyme sites (CA1), following a previously reported cloning strategy.[2] These restriction sites were chosen in order to remove a N-terminal signal peptide and a C-terminal histidine tag present in the original vector, so that the expression vectors obtained with this strategy encode the native protein sequences. Specifically, CA2 was amplified using the following primers:

Forward: 5'-CCGGAATTCGCCACCATGGCCCATCACTGGGGGTACGGC-3';

Reverse: 5'-CCGCTCGAGTTATTTGAAGGAAGCTTTGATTTGCCTGTTCTTC-3';

CA1 was amplified using the following primers:

Forward: 5'-CCCAAGCTTGCCACCATGGCAAGTCCAGACTGGGGATATGATG-3';

Reverse: 5'-CCGCTCGAGTCAAAATGAAGCTCTCACTGTTCTGCCCTTC-3'.

A Kozak sequence was inserted in both forward primers downstream of the EcoRI/HindIII site, while a stop codon was inserted in both reverse primers upstream of the XhoI site. The clones were verified by DNA sequencing.

1.2. Human cell culture and transfection

HEK293T (ATCC CRL-3216) cells were maintained in Dulbecco-modified Eagle medium (DMEM) high glucose (Gibco) supplemented with L-glutamine, antibiotics (penicillin and streptomycin) and 10% fetal bovine serum (FBS, Gibco) in uncoated 75 cm^2 plastic flasks and incubated at 37 °C, 5% CO2 in a humidified atmosphere. Cells were transiently transfected with the pHLsec plasmid containing the gene of CA2 or CA1 using polyethylenimine (PEI), with a DNA:PEI ratio of 1:2 (25 µg/flask DNA, 50 µg/flask PEI). Lower expression levels of CA2 were obtained by transfecting cells with a mixture of 5 µg CA2 DNA and 20 µg empty vector. DMEM medium was used for unlabelled in-cell NMR samples; [U-^{15}N]-BioExpress6000 medium (Cambridge Isotope Laboratories) was used for uniform ^{15}N in-cell NMR samples. Expression media were supplemented with 2% FBS, antibiotics and 10 µM $ZnSO_4$.

1.3. Protein quantification and intracellular distribution

The expression level of CA2 was determined by Coomassie-stained SDS-PAGE. Densitometry analysis was performed with ImageJ. Lysates from cell samples were run at increasing dilutions together with

purified CA2 as a reference. CA2 expression at lower levels was assessed by SDS-PAGE of lysates from cells transfected with lower amounts of CA2 DNA (Figure S12a). CA1 level was estimated by comparison with CA2 (Figure S12b). The values reported in the main text reflect the protein concentrations calculated from cells lysed in 1 cell pellet-volume, therefore corresponding to the effective concentrations in the in-cell NMR samples (mean value ± s.d., n =3). Intracellular distribution was assessed by separating the insoluble fraction (containing the nuclei and other membranous fractions), the cytosolic and the mitochondrial fraction from cell extracts using a mitochondria isolation kit for cultured cells (Thermo Fisher Scientific), followed by analysis via SDS-PAGE of each fraction at serial dilutions. Following the separation, >90% of CA2 was recovered in the cytosolic fraction, whereas <5% was recovered in the insoluble fraction and <2% was recovered in the mitochondrial fraction (Figure S12d).

1.4. Treatment with inhibitors

HEK293T cells overexpressing CA2 or CA1 were treated with each inhibitor 48 hours post-transfection. Dose-dependent binding curves were obtained by treating different cell cultures with increasing amounts of each ligand for a fixed time (1 hour, unless specified differently in the main text). Time-dependent binding curves were obtained by treating different cell cultures with a fixed amount of AAZ or MZA for increasing amounts of time. The cellular response to AAZ and MZA in terms of cell viability was assessed by trypan blue staining of cells overexpressing CA2 and subsequently treated for 1 hour with increasing concentrations of AAZ or MZA (0, 1, 10, 100 μM). Viability remained > 95% in all samples (Figure S12c).

1.5. Stopped-flow measurements

An Applied Photophysics stopped-flow instrument was used for assaying the CA2-catalysed CO_2 hydration activity.[3] CA2 was purchased from Sigma-Aldrich. Phenol red (at a concentration of 0.2 mM) was used as indicator, working at the absorbance maximum of 557 nm, with 20 mM HEPES (pH 7.5) as buffer, and 20 mM Na_2SO_4 (for maintaining constant the ionic strength), following the initial rates of the CA2-catalysed CO_2 hydration reaction for a period of 10–100 s. The CO_2 concentrations ranged from 1.7 to 17 mM for the determination of the kinetic parameters and inhibition constants. For each inhibitor at least six traces of the initial 5–10% of the reaction have been used for determining the initial velocity. The uncatalyzed rates were determined in the same manner and subtracted from the total observed rates. Stock solutions of inhibitor (0.1 mM) were prepared in distilled-deionized water and dilutions up to 0.01 nM were done thereafter with the assay buffer. Inhibitor and enzyme solutions were preincubated together for 15 min at room temperature prior to assay, in order to allow for the formation of the E-I complex. The inhibition constants were obtained by non-linear least-squares methods using PRISM 3 and the Cheng–Prusoff equation and represent the mean (± s.d.) from at least three different determinations.

Cell lysates were prepared by freeze-thaw cycles in phosphate buffered saline (PBS), pH 7.4 followed by centrifugation for 1 hour at 16000 g, 4°C. The supernatant from each sample was collected. Total protein concentration in each sample was measured by BCA Protein Assay (Thermo Fisher), and the sample volumes were adjusted with PBS to equalize total protein concentration, and subsequently diluted 400-fold. Total CO_2 hydration activity in the cell lysates was measured by stopped-flow using the same protocol described above.

1.6. Expression and purification of recombinant CA2

Recombinant CA2 for in vitro experiments was prepared following an existing protocol.[4] Briefly, a 1-liter cell culture of *E. Coli* BL21(DE3) Codon Plus Ripl (Stratagene) transformed with a pCAM plasmid containing the CA2 gene was grown overnight at 37°C in LB, harvested and re-suspended in 1 liter of ^{15}N-labelled M9 medium. $ZnSO_4$ was added in the culture to a final concentration of 500 µM. After 5 h from induction with 1 mM IPTG at 37°C the cells were harvested and re-suspended in 20 mM Tris, pH 8 buffer for lysis. The cleared lysate was loaded onto a nickel chelating HisTrap (GE Healthcare) 5 ml column. The protein was eluted with a linear gradient of 20 mM Tris pH 8, 500 mM imidazole. The fractions containing pure CA2 were collected. Finally, the protein was exchanged in NMR buffer (PBS pH 7.4, Gibco, supplemented with 10% D_2O). The correct metalation of the protein was confirmed by chemical shift comparison against previously reported spectra.

1.7. In vitro NMR experiments

Samples of pure CA2 (170 µM in NMR buffer), both unlabelled and ^{15}N-labelled, were placed in 5 mm NMR tubes and analyzed at 310 K at a 900 MHz Bruker Avance HD spectrometer equipped with a TCI CryoProbe. 1D ^{1}H WATERGATE 3-9-19 (on the unlabelled protein) and 2D ^{1}H-^{15}N SOFAST-HMQC (on the ^{15}N-labelled protein) spectra were acquired both in the absence of ligands and upon addition of 1 equivalent of each ligand. The same CA2 samples were then titrated with up to 2 equivalents of each ligand and analyzed by NMR to exclude the presence of high-affinity secondary binding sites. For some ligands, poor solubility in aqueous buffer caused precipitate formation, without affecting protein solubility. In those circumstances, complete binding was obtained by adding step-wise 2-3 equivalents to the protein, spinning down after each addition to remove the precipitate.

1.8. In-cell NMR experiments

Samples for in-cell NMR were prepared following a reported protocol.[2] Briefly, cells overexpressing CA2/CA1 were detached with trypsin, suspended in DMEM + 10% FBS, washed once with PBS and re-suspended in one pellet volume of DMEM supplemented with 90 mM glucose, 70 mM HEPES and 20% D_2O. The cell suspension was transferred in a 3 mm Shigemi NMR tube, which was gently spun to sediment the cells. Cell viability before and after NMR experiments was assessed by trypan blue staining. In-cell NMR spectra were collected at 310 K at a 900 MHz Bruker Avance HD spectrometer

equipped with a TCI CryoProbe. 2D ^{1}H-^{15}N SOFAST-HMQC spectra were recorded on ^{15}N-labelled cell samples; 1D ^{1}H WATERGATE 3-9-19 spectra were recorded on unlabelled cell samples. The acquisition time for cells expressing CA2 at high levels was ~1 hour for 2D NMR spectra and ~30 minutes for 1D NMR spectra. 3-hours long 1D NMR spectra were recorded on unlabelled cells expressing either CA2 at lower levels or CA1. To preserve cell viability, two identical cell samples for each condition were analyzed for 1.5 hour each and the obtained spectra were accumulated during processing. After the NMR experiments, the cells were collected and the supernatant was checked for protein leakage by NMR.

1.9. NMR data processing and signal deconvolution

The NMR spectra were acquired and processed with Bruker Topspin software. The 2D in-cell NMR spectra of ^{15}N-labelled cells were further processed by subtracting an identical spectrum recorded on cells transfected with empty vector, acquired in the same experimental conditions, to eliminate the signals arising from partial ^{15}N incorporation in other cellular components. Backbone amide resonances were assigned based on available data[5]. Combined chemical shift difference (CCSD) for each amide crosspeak was calculated using the formula:

$$CCSD = \sqrt{\frac{\left(\delta\Delta\ ^{1}H\right)^{2} + \left(\delta\Delta^{15}N/5\right)^{2}}{2}}$$

Signals in the 1D ^{1}H in-cell NMR spectra were deconvoluted using Fityk software.[6] The spectral region between 11 and 16 ppm was isolated and globally fitted with a sum of N+1 pseudo-Voigt functions (with gaussian weight fixed at 0.15), where N equals the number of discernible signals in the region. The additional function accounted for baseline distortions caused by strong signals outside the spectral region. Relative signal intensities were obtained as the ratio of the area under each fitted peak and the sum of the N areas. Signals that were not perturbed by ligand binding or could not be separated due to high overlap were discarded. Signal intensities were plotted against ligand concentration or time to obtain dose- and time-dependent binding curves, respectively.

1.10. Binding curve fitting

The binding curves were fitted with either dose- and time-dependent or dose-dependent, time-independent binding equations using OriginPro software. Equations were obtained from a model accounting for passive diffusion of the ligand through the plasma membrane: $L_o \rightleftarrows L_i$, followed by binding to the intracellular protein: $L_i + P \rightleftarrows LP$, where L_o and L_i are the extracellular and intracellular ligand, respectively, and P and LP are the free and the bound protein, respectively. Passive diffusion was considered the rate-determining step, with the rate equation:

$$\frac{d[L_{t.in}]}{dt} = \frac{d([L_i] + [LP])}{dt} = \frac{K_p A}{V_c}([L_o] - [L_i])$$

where $[L_{t.in}]$ is the total intracellular ligand concentration, K_p is the permeability coefficient, A is the total area of the membrane and V_c is the intracellular volume. Free and bound protein were considered to reach the equilibrium instantly at each value of $[L_{t.in}]$, therefore the bound protein [LP] was obtained from the equilibrium binding equation:

$$[LP](t) = \frac{K_d + [P_t] + [L_{t.in}](t) - \sqrt{\left(K_d + [P_t] + [L_{t.in}](t)\right)^2 - 4[P_t][L_{t.in}](t)}}{2}$$

where $[P_t]$ is the total intracellular protein concentration and K_d is the dissociation constant. The above equation was used to fit both the dose-dependent and the time-dependent binding curves (rate-limited by membrane diffusion), to obtain values of $K_p \cdot A$, by expressing $[L_{t.in}](t)$ with the integral diffusion rate equation approximated for $[L_i] << [L_o]$:

$$L_{t.in}(t) = L_t\left(1 - e^{-\frac{K_p A}{V_t}t}\right)$$

where $L_t = L_o + L_i + LP$ are the total moles of ligand. Time-dependent curves in defect of external ligand were fitted with a simplified model where the free intracellular ligand $[L_i]$ is considered in steady-state and at negligible concentration, and LP(t) is directly obtained from the membrane diffusion rate:

$$\frac{d[LP]}{dt} = \frac{K_p A}{V_c}([L_o] - [L_i])\ ;\ LP(t) = L_t\left(1 - e^{-\frac{K_p A}{V_t}t}\right)$$

Finally, to obtain an apparent K_d, dose-dependent curves obtained at longer times were fitted with the time-independent binding equilibrium equation calculated for $[L_o] = [L_i] = [L]$:

$$[LP] = \frac{K_d + [P_t]V_r + [L_t] - \sqrt{(K_d + [P_t]V_r + [L_t])^2 - 4[P_t][L_t]V_r}}{2V_r}$$

where $V_r = V_c/V_t$ is the cell/total volume ratio and $[L_t] = [L] + [LP]V_r$.

References

[1] A. R. Aricescu, W. Lu, E. Y. Jones, *Acta Crystallogr. D Biol. Crystallogr.* **2006**, *62*, 1243–1250.
[2] L. Barbieri, E. Luchinat, L. Banci, *Nat. Protoc.* **2016**, *11*, 1101–1111.
[3] R. G. Khalifah, *J. Biol. Chem.* **1971**, *246*, 2561–2573.
[4] L. Cerofolini, S. Giuntini, A. Louka, E. Ravera, M. Fragai, C. Luchinat, *J. Phys. Chem. B* **2017**, *121*, 8094–8101.
[5] W. L. Nettles, H. Song, E. R. Farquhar, N. C. Fitzkee, J. P. Emerson, *Inorg. Chem.* **2015**, *54*, 5671–5680.
[6] M. Wojdyr, *J. Appl. Crystallogr.* **2010**, *43*, 1126–1128.
[7] H. Shimahara, T. Yoshida, Y. Shibata, M. Shimizu, Y. Kyogoku, F. Sakiyama, T. Nakazawa, S. Tate, S. Ohki, T. Kato, et al., *J. Biol. Chem.* **2007**, *282*, 9646–9656.
[8] S. K. Vasa, H. Singh, K. Grohe, R. Linser, *Angew. Chem. Int. Ed Engl.* **2019**, *58*, 5758–5762.

2. Supplementary Figures

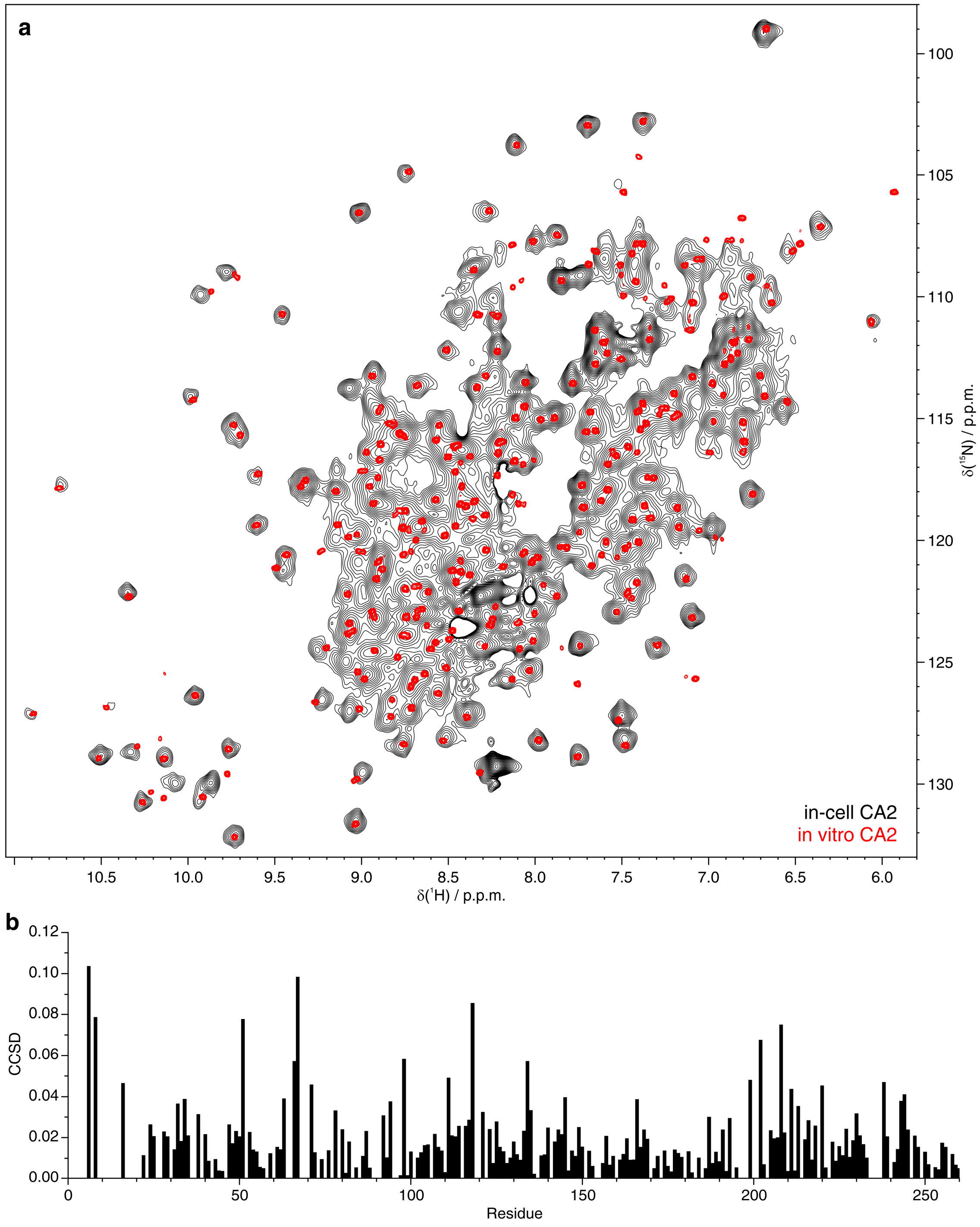

Figure S1. (**a**) Overlay of ^{1}H-^{15}N NMR spectra of cells expressing CA2 (black) and in vitro CA2 (175 μM, red). (**b**) Combined ^{1}H-^{15}N chemical shift difference (CCSD) between in vitro and in-cell CA2.

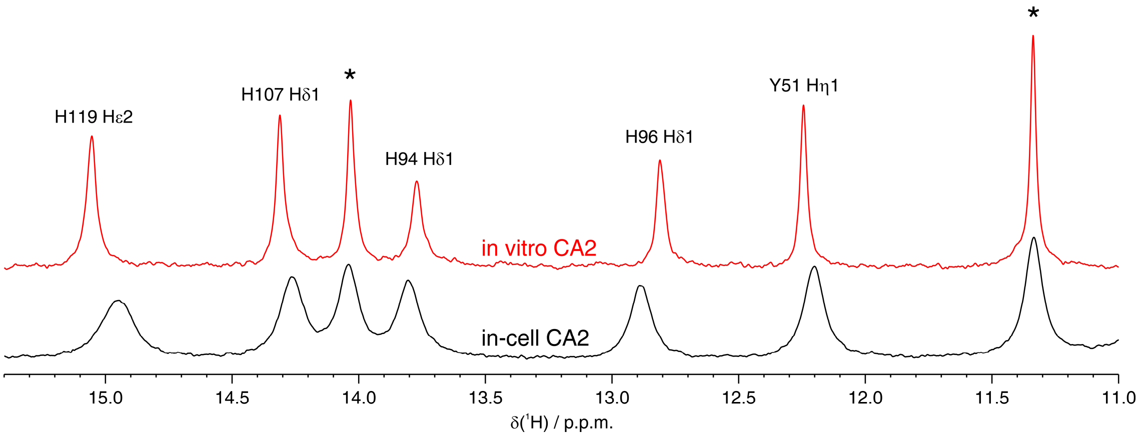

Figure S2. Imino region of the 1D ^{1}H NMR spectrum of CA2 in vitro (red) and in-cell (black). Signals for which the unambiguous assignment has been reported previously are labelled with the corresponding residue number and atom type.[7,8] Signals arising from unassigned protons are labelled with an asterisk. H94, H96 and H119 are the zinc-coordinating histidines in the active site of CA2.

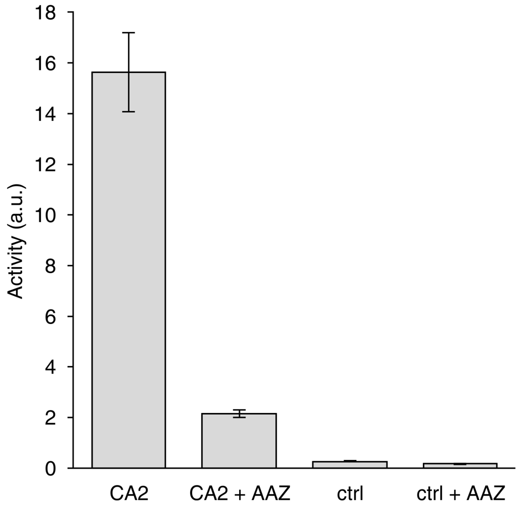

Figure S3. Total CO_2 hydrase activity measured on lysates from HEK293T cells overexpressing CA2 (CA2) and control cells with basal levels of CA2 (ctrl), either untreated or treated with 100 μM AAZ for 1 hour. Error bars represent s.d. (n = 3 independent samples).

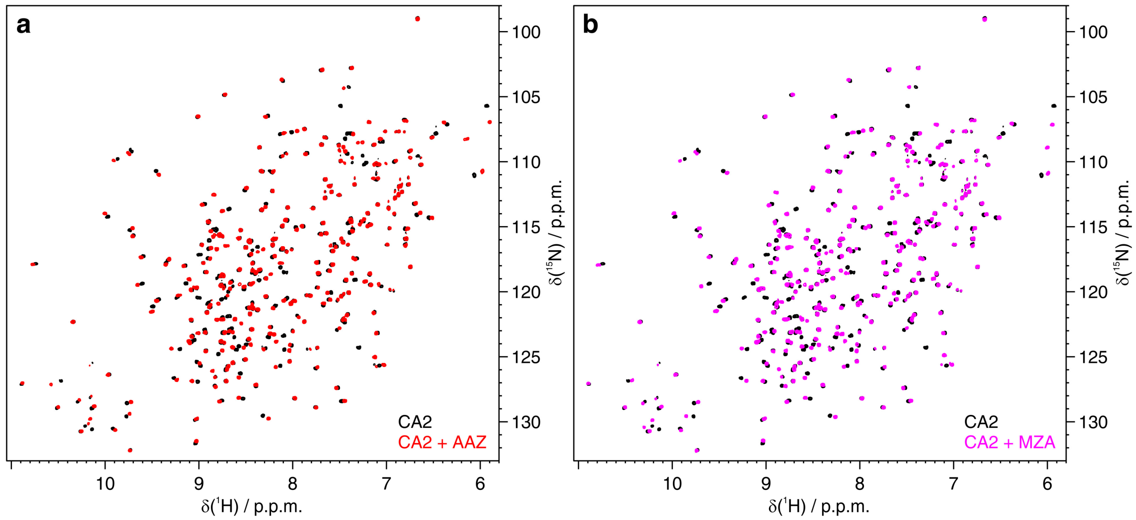

Figure S4. Overlay of ^{1}H-^{15}N NMR spectra of CA2 in vitro (175 μM) in the absence of ligands (black) and in the presence of 1 equivalent of (**a**) AAZ (red) or (**b**) MZA (magenta). The appearance of new sets of peaks in the presence of AAZ/MZA together with the complete disappearance of signals arising from free CA2 indicate quantitative ligand binding.

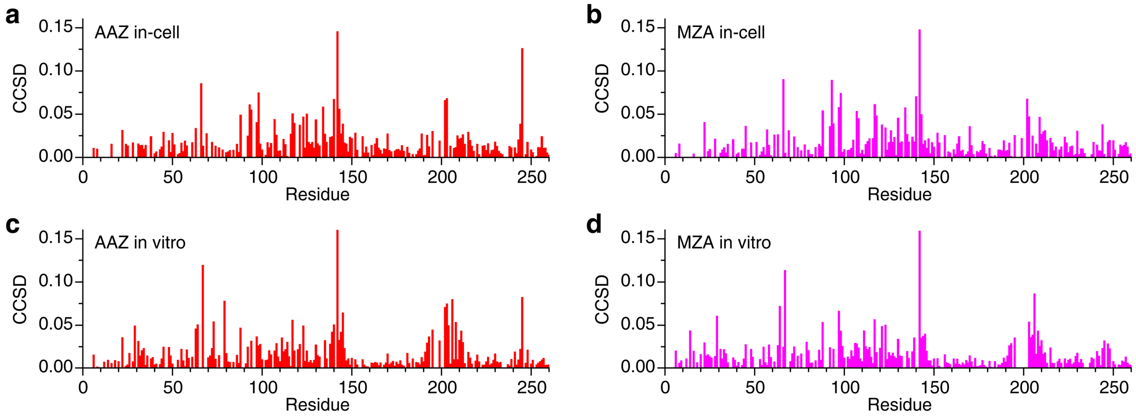

Figure S5. (**a, b**) in-cell ^{1}H-^{15}N CCSD between CA2 in the absence of ligands and bound to (**a**) AAZ; (**b**) MZA. (**c, d**) in vitro ^{1}H-^{15}N CCSD between CA2 in the absence of ligands and bound to (**c**) AAZ; (**d**) MZA.

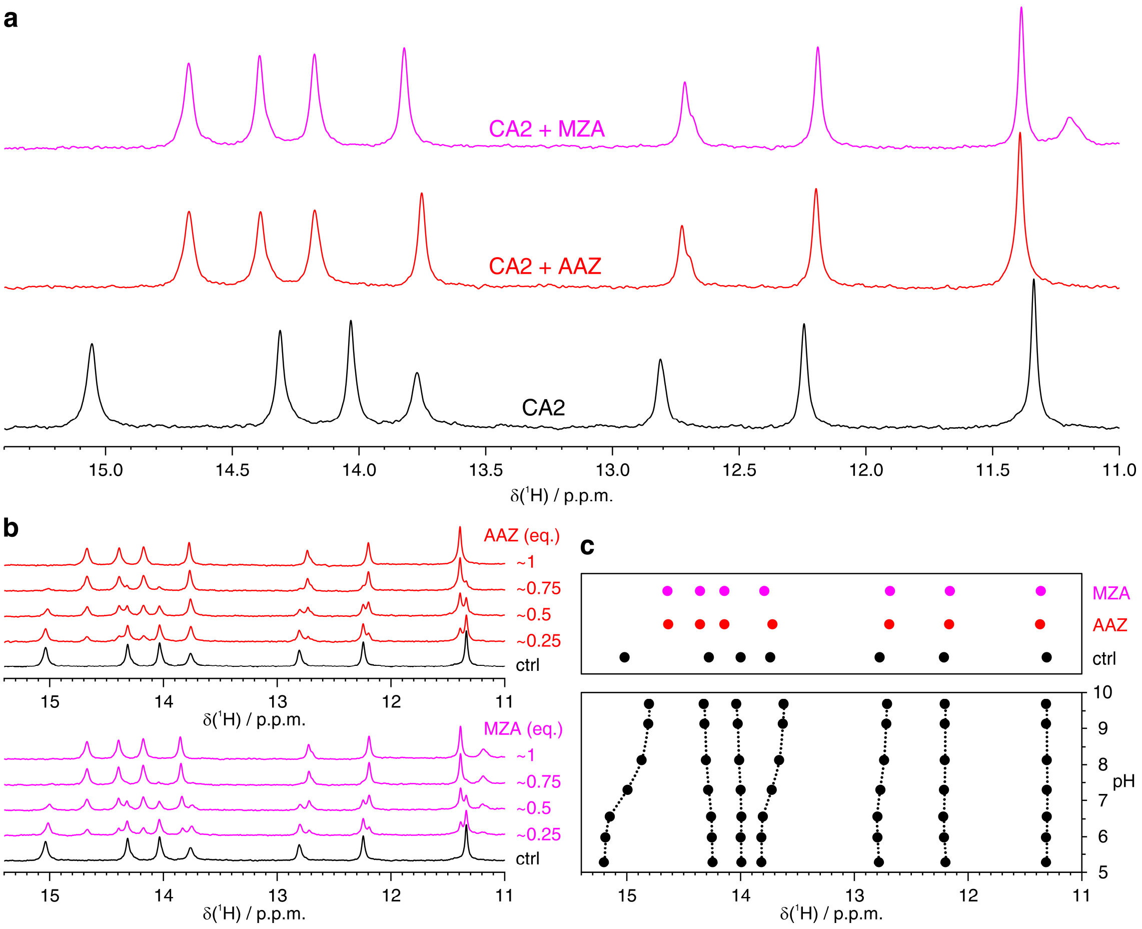

Figure S6. (**a**) Imino region of the 1D ^{1}H NMR spectra of in vitro CA2 in the absence of ligands (black) and bound to 1 equivalent of AAZ (red) or MZA (magenta). (**b**) 1D ^{1}H NMR spectra of in vitro CA2 in the absence of ligands (black) and upon addition of sub-stoichiometric amounts of AAZ (red) or MZA (magenta). Peaks arising from both free and bound CA2 are observed simultaneously. (**c**) Plot of the ^{1}H chemical shifts observed in the same spectral region as in (**a**) and (**b**) in the in vitro NMR spectra of free CA2 (black), AAZ-bound CA2 (red) and MZA-bound CA2 (magenta), measured at pH 7.4, compared to the ^{1}H chemical shift plot of free CA2 as a function of pH.

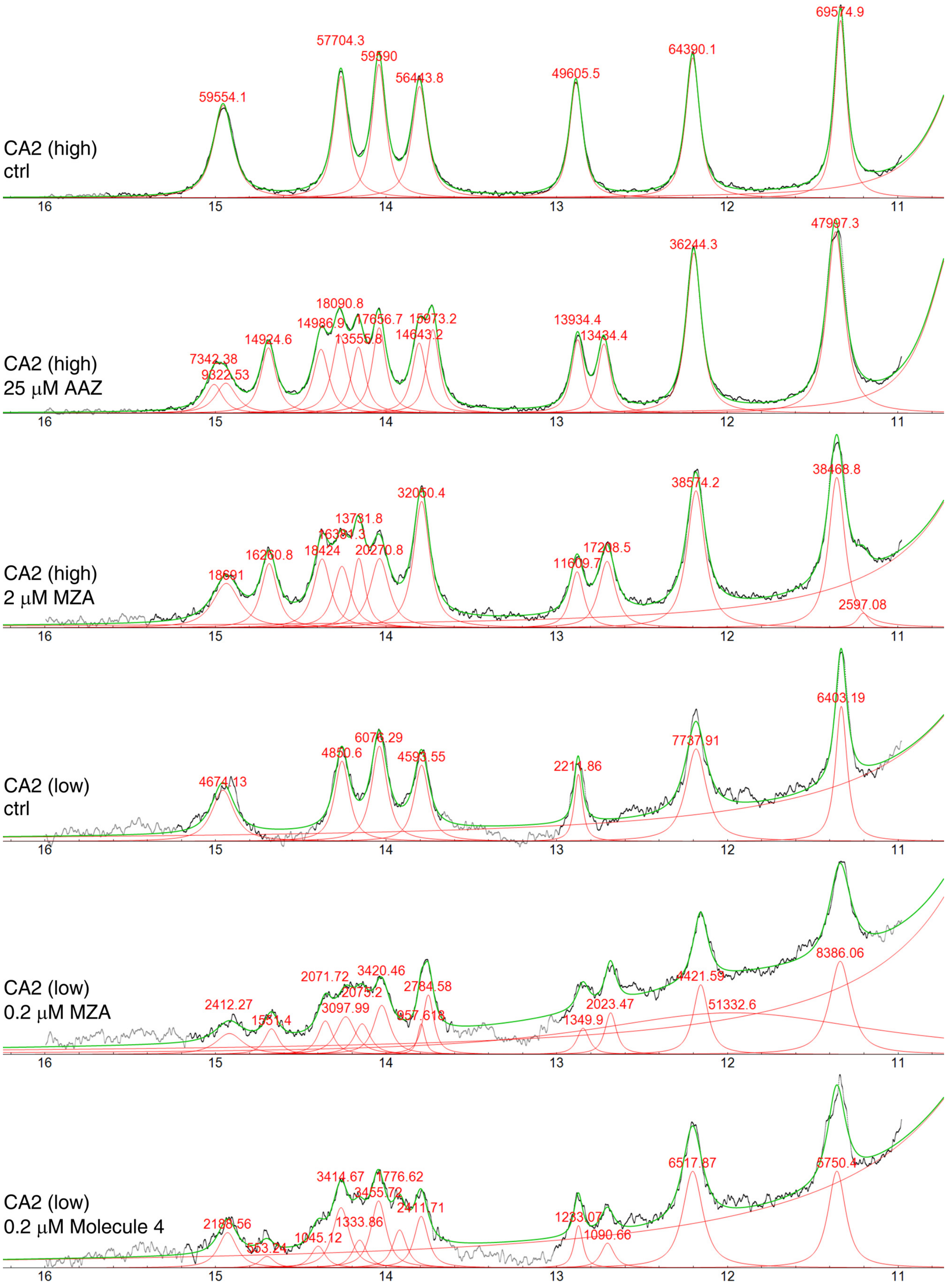

CA2 (high)
ctrl
59554.1
57704.3
59590
56443.8
49605.5
64390.1
69574.9
CA2 (high)
25 μM AAZ
7342.38
9322.53
14924.6
14986.9
18090.8
13555.8
17656.7
15973.2
14643.2
13934.4
13434.4
36244.3
47997.3
CA2 (high)
2 μM MZA
18691
16260.8
18424
13731.8
20270.8
32050.4
11609.7
17208.5
38574.2
38468.8
2597.08
CA2 (low)
ctrl
4674.13
4850.6
6076.29
4593.55
2211.86
7737.91
6403.19
CA2 (low)
0.2 μM MZA
2412.27
2071.72
3420.46
3097.99
2784.58
2023.47
1349.9
4421.59
51332.6
8386.06
CA2 (low)
0.2 μM Molecule 4
2188.56
563.24
1045.12
3414.67
1333.86
1776.62
3455.72
2411.71
1233.07
1090.66
6517.87
5750.4
16
15
14
13
12
11

Figure S7. Spectral deconvolution in the imino region of different in-cell NMR spectra expressing CA2 at high (~150 µM) or low (~45 µM) levels. Both untreated cells (ctrl) and cells treated with concentrations of ligands that result in the incomplete binding are shown. The raw NMR data (black), single peak Pseudo-Voigt functions (red) and the overall sum (green) are shown. Calculated peak areas are indicated in red. In some instances, peaks with non-ideal shapes were fitted as the sum of two Pseudo-Voigt functions. Additional functions were used to correct for baseline distortions, the area of which was not used in the normalization process.

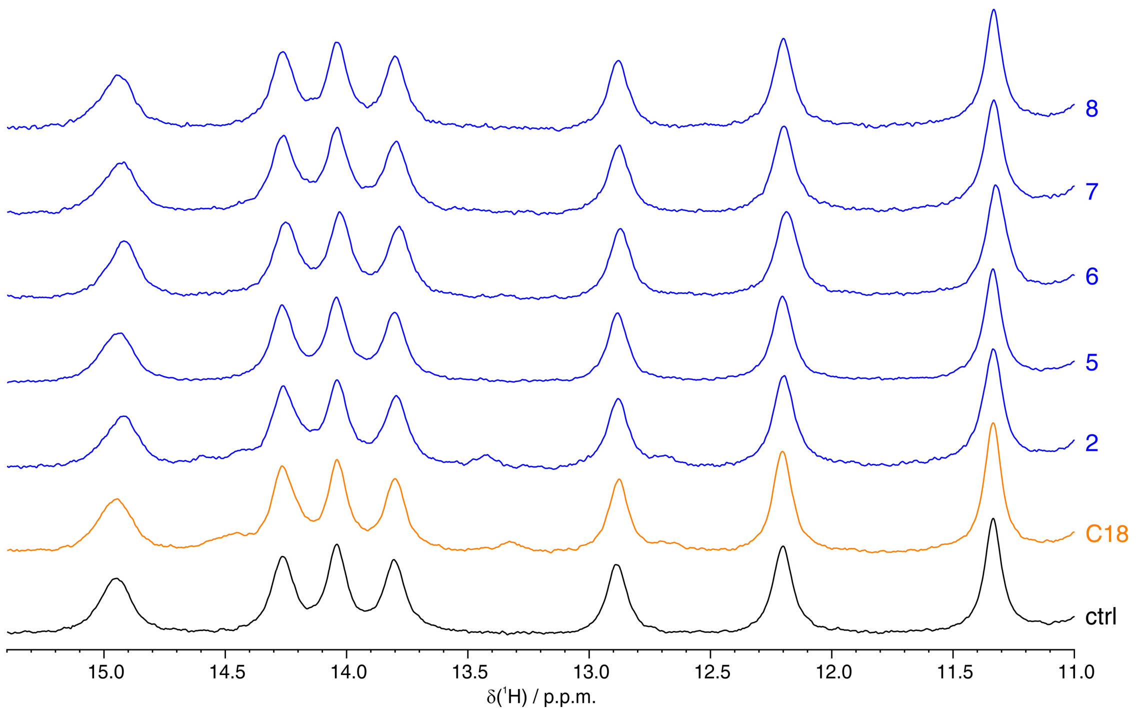

Figure S8. Imino region of the 1D ^{1}H NMR spectra of cells expressing CA2 in the absence of ligands (black) and treated with ligands showing negligible binding to intracellular CA2: C18 (orange), 2, 5, 6, 7 and 8 (blue).

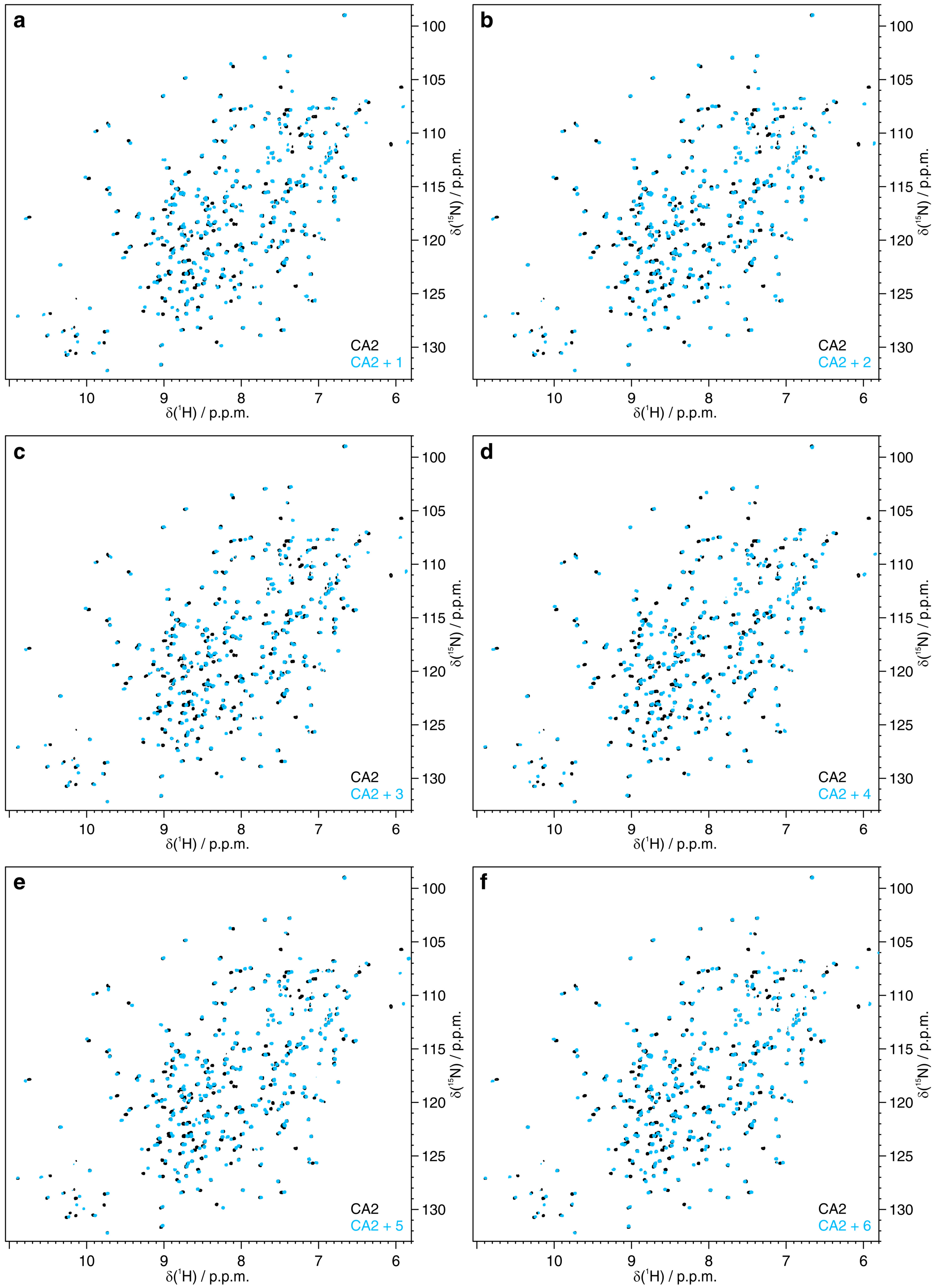
a
CA2
CA2 + 1
b
CA2
CA2 + 2
c
CA2
CA2 + 3
d
CA2
CA2 + 4
e
CA2
CA2 + 5
f
CA2
CA2 + 6
δ(¹H) / p.p.m.
δ(¹⁵N) / p.p.m.
10
9
8
7
6
100
105
110
115
120
125
130

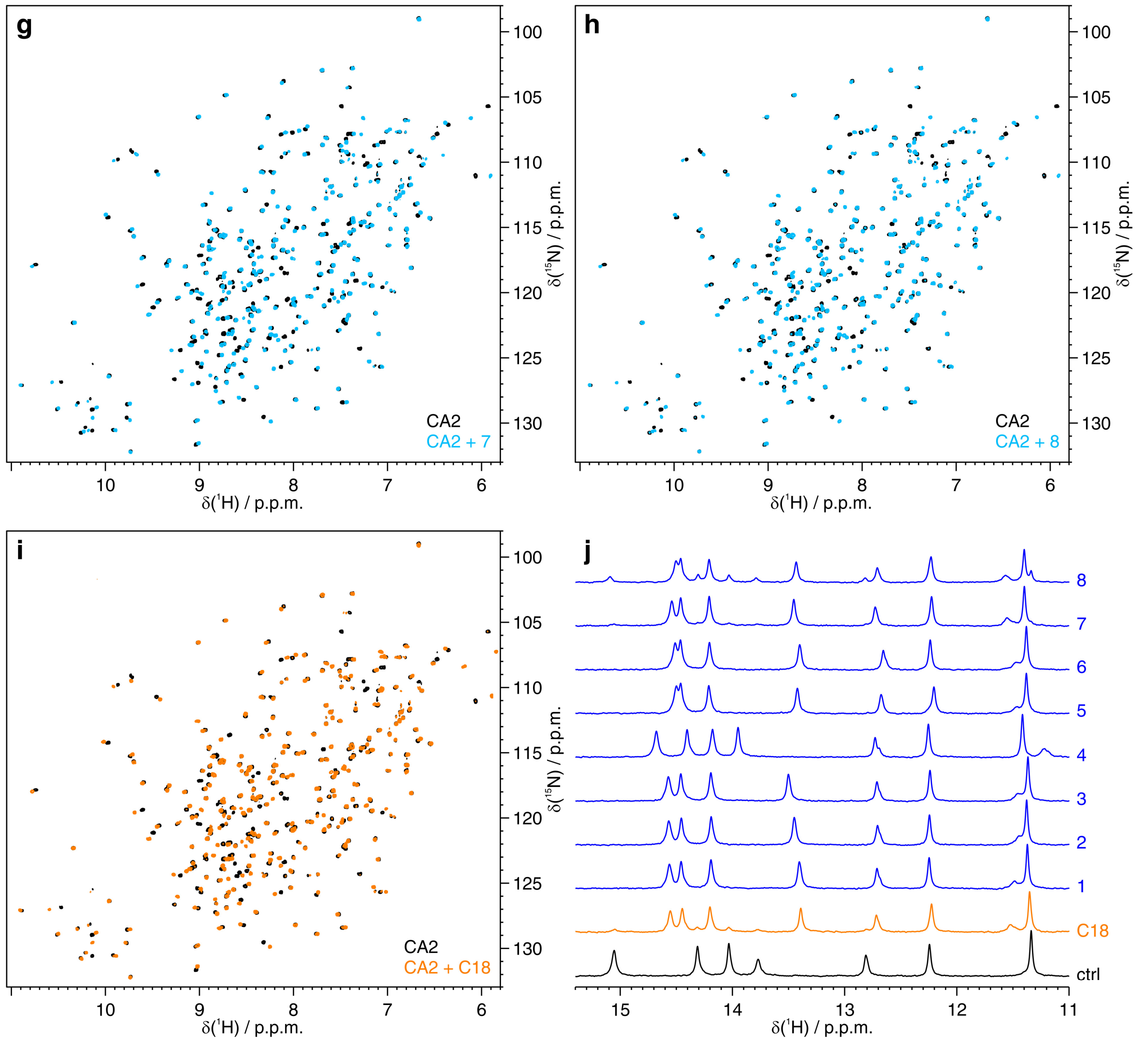

Figure S9. Overlay of ^{1}H-^{15}N NMR spectra of CA2 in vitro (175 µM) in the absence of ligands (black) and in the presence of 1 equivalent of (**a-h**) ligands 1 to 8 (light blue) or (**i**) C18 (orange). (**j**) Imino region of the 1D ^{1}H NMR spectra of CA2 in vitro in the absence of ligands (black) and bound to ligands 1 to 8 (blue) or C18 (orange). Complete binding was not observed for the lowest-affinity ligand, 8, likely due to its precipitation in aqueous buffer at the concentrations required.

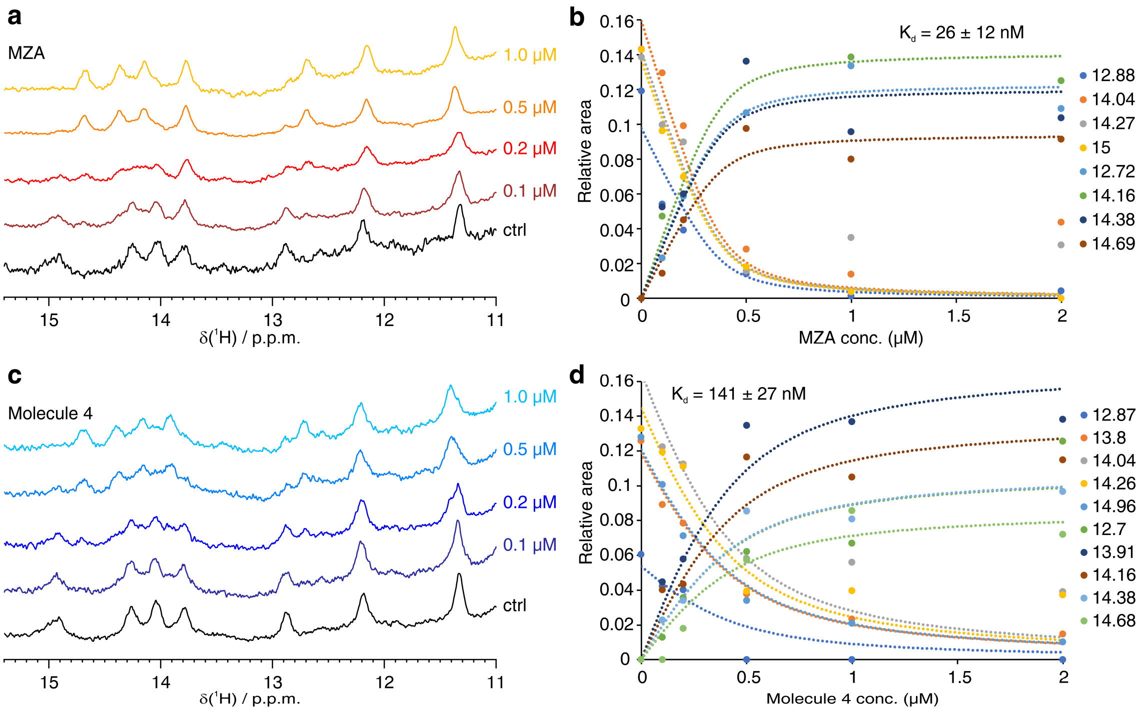

Figure S10. (**a, c**) Imino region of the 1D ^{1}H NMR spectra of cells expressing CA2 at lower levels (~45 µM in the NMR tube) in the absence of ligands (black) and treated for 2 hours with increasing concentrations of MZA (**a**, brown to yellow) or Molecule 4 (**c**, dark blue to cyan) at increasing concentrations. (**b, d**) Dose-dependent binding curves obtained from the NMR spectra shown in (**a**) and (**c**), respectively, fitted with a time-independent binding equation (see Experimental Methods). Binding of MZA reaches 50% at around 0.3 µM, giving an apparent K_d of 26 ± 12 nM (**b**), close to the K_d measured in vitro (14 ± 0.9 nM). Instead, binding of Molecule 4 requires a higher dose, giving an apparent K_d of 141 ± 27 nM (**d**), ~20-fold higher than in vitro (5.9 ± 0.4 nM), which suggests the presence of competing binding sites within the cell.

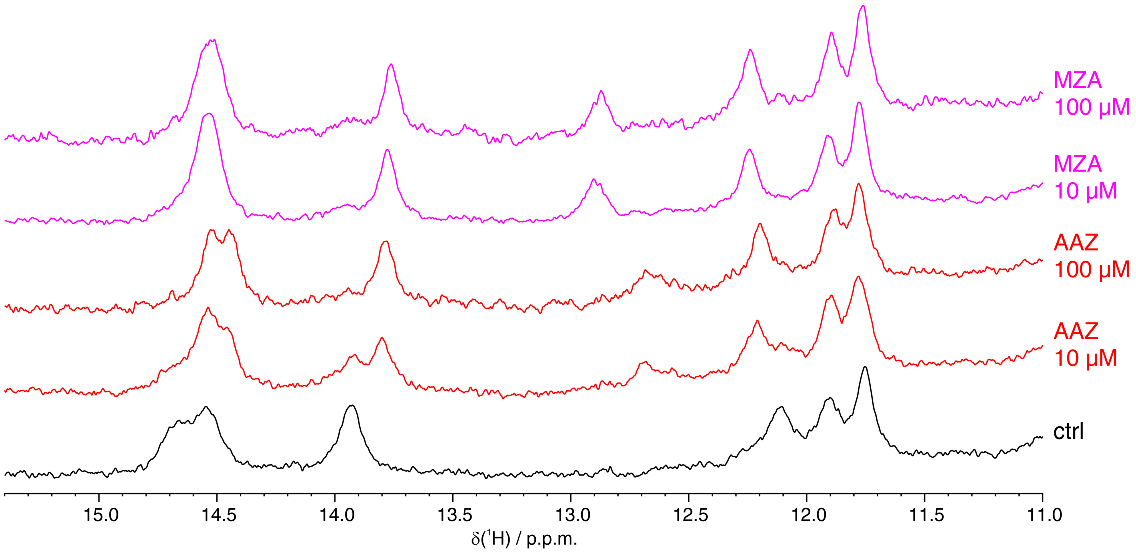

Figure S11. Imino region of the 1D ^{1}H NMR spectra of cells expressing CA1 (~50 µM in the NMR sample) in the absence of ligands (black) and in the presence of AAZ 10 µM and 100 µM (red), or MZA 10 µM and 100 µM (magenta).

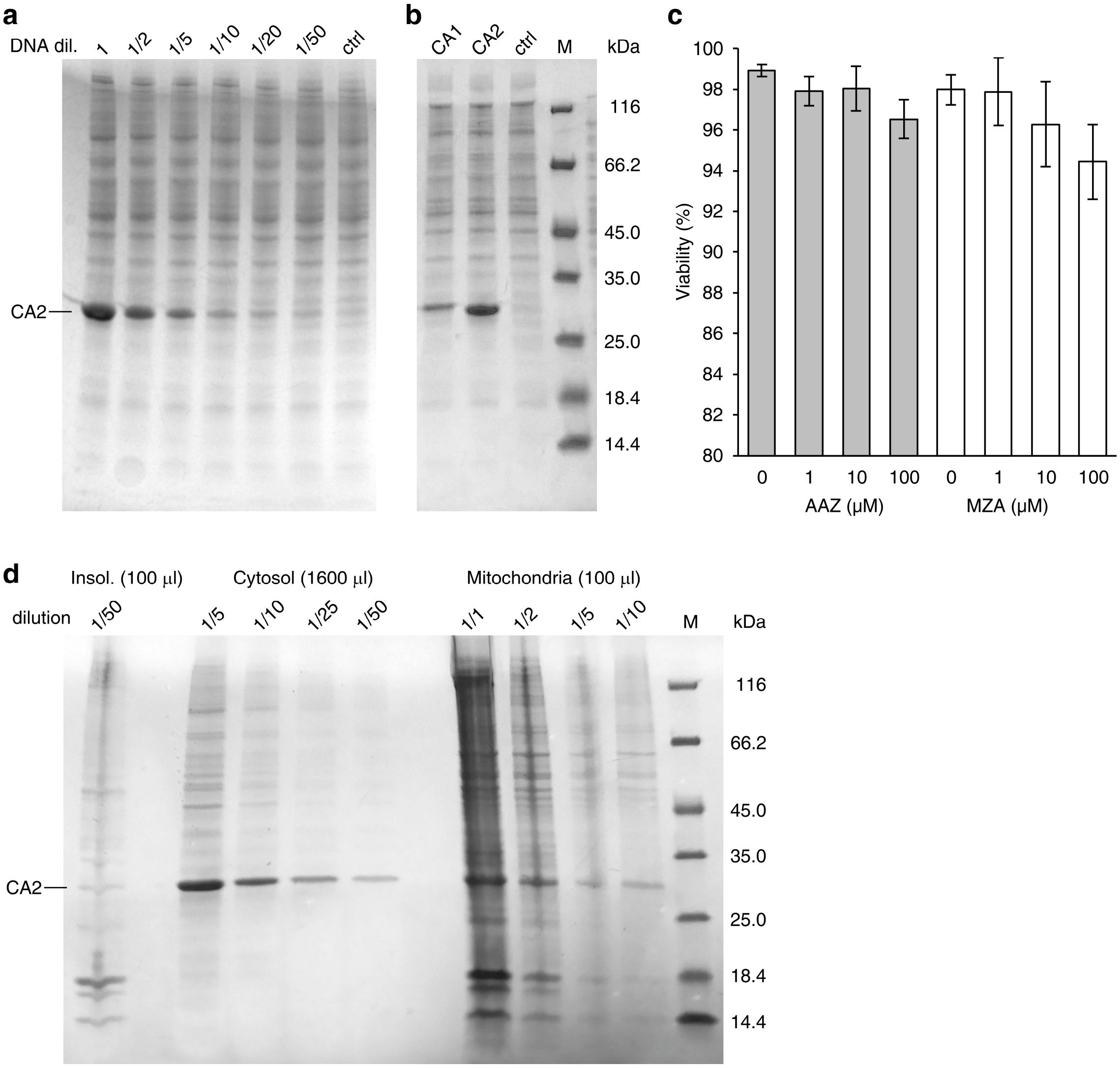

Figure S12. (**a**) Coomassie-stained SDS-PAGE of cleared lysates from cells transfected with the vector containing the CA2 gene at different dilutions with empty vector. Protein concentration was estimated by densitometry analysis with respect to a sample of recombinant CA2 at known concentration (not shown); (**b**) Coomassie-stained SDS-PAGE comparing lysates from cells expressing CA1 and CA2. M = marker; (**c**) cell viability measured by Trypan blue on cells treated for 1 hour with increasing concentration of AAZ (grey bars) or MZA (white bars). Error bars represent standard deviation (n = 3). (**d**) Intracellular distribution of overexpressed CA2. Insol.: insoluble fraction containing nuclei and cell membranes. For each fraction, the total volume and the loading dilution are indicated. M = marker.
